# THE LONG-TERM EFFECTS OF EARLY SENSORY IMPAIRMENTS ON LATER-LIFE DISABILITY AND LONELINESS

**DOI:** 10.1093/geroni/igae098.1809

**Published:** 2024-12-31

**Authors:** Eunbea Kim, Gina Lee, Peter Martin, JeongEun Lee

**Affiliations:** Iowa State University, Ames, Iowa, United States; University of Wisconsin Madison, Madison, Wisconsin, United States; Iowa State University, Ames, Iowa, United States; Iowa State University, Ames, Iowa, United States

## Abstract

We focus on the long-term consequences of childhood sensory impairments on physical disability and loneliness in later life, examining the mediating roles of childhood parental relationships in the association between childhood sensory impairments and later life sensory difficulties. We further investigated whether childhood socioeconomic factors (i.e., family income and parents’ educational attainment) moderate these relationships. Data from the Health and Retirement Study (HRS; 2012 and 2016; n = 2,116, Mage = 71.47), combined with the HRS Life History data, was utilized. Using path analyses in R, the analysis revealed that childhood vision impairment was related to higher late-life physical disability and loneliness through higher vision difficulties in late adulthood. These relationships were not influenced by the moderators (family income and parents’ educational attainment). Conversely, early hearing impairment was linked to greater older adulthood loneliness, mediated by poor childhood parental relationships. The negative impact of poor parental relationships on loneliness was lessened with a higher childhood family income and parental education for those with childhood hearing problems. Notably, mothers’ higher education level significantly alleviates the risk of later-life hearing difficulties for those with childhood hearing problems. This research underlines the lasting effects of childhood experiences and parents’ background on well-being in later life as well as the different consequences of vision and hearing difficulties. These findings shed light on the understanding of how childhood experiences can shape disability and loneliness later in life, underscoring important implications for prevention efforts in public health and policy.

